# Configurations for positive public behaviors in response to the COVID-19 pandemic: a fuzzy set qualitative comparative analysis

**DOI:** 10.1186/s12889-022-14097-6

**Published:** 2022-09-06

**Authors:** Junwang Gu, Chunmei Wu, Xuanhui Wu, Rong He, Jing Tao, Wenhui Ye, Ping Wu, Ming Hao, Wei Qiu

**Affiliations:** School of Public Health and Health Management, Gannan Medical University, Ganzhou, 341000 Jiangxi Province China

**Keywords:** Emergency risk management, Configurations, Public behaviors, Fuzzy set qualitative comparative analysis

## Abstract

**Background:**

The COVID-19 crisis poses considerable threats to public health, and exploring the key configuration conditions of the public behavior response is very important for emergency risk management.

**Objective:**

This study attempts to reveal differences in the conditional configuration and mechanism of public behavior based on the proposed framework, further make up for the deficiencies of existing research in explaining such issues as “How to promote the public’s protective behavior or reduce the public’s excessive behavior?” and finally provide new evidence and ideas for the government to improve the emergency management system.

**Methods:**

A total of 735 valid cases were obtained using an online survey and revealed the conditional configuration and mechanism of public behavior differences through a fuzzy set qualitative comparative analysis based on the proposed public behavioral framework.

**Results:**

The results show that critical factors including risk communication, trust, risk perception, and negative emotions alone did not constitute a necessary condition for public protective or excessive behavior. The different configurations of influencing factors reveal the complexity of public behavioral risk management, and taking adequate measures to increase public trust and reduce negative public emotions constitute the core path of risk management to enhance positive public behavior.

**Conclusions:**

The configurations of various influencing factors reveal the complexity of public behavioral risk management. For behavioral risk management, governments should focus on adapting to multiple conditions according to their situations and, under the “overall perspective,” formulate policies based on local conditions and further form a differentiated risk management path. Practically speaking, for the government, taking adequate measures to increase public trust and reduce negative public emotions is the core path of risk management to enhance positive public behavior.

**Supplementary Information:**

The online version contains supplementary material available at 10.1186/s12889-022-14097-6.

## Introduction

The Coronavirus disease 2019 (COVID-19) pandemic has posed considerable threats to public health and life worldwide since its outbreak in 2019 [1]. Since 2020, COVID-19 has undergone multiple mutations from the original strain to the Delta and Omicron variants, and its transmission capacity has continued to increase. Therefore, the threat of COVID-19 remains ongoing. Globally, as of 4 April 2022, there have been 489,779,062 confirmed cases of COVID-19, including 6,152,095 deaths; according to the World Health Organization mortality has showed an upward trend [2]. The pandemic continues to manifest in China and many other countries, which poses enormous challenges to the resilience of the global public health system [3, 4].

To combat the COVID-19 pandemic, various governments have adopted different epidemic prevention and control strategies according to their national conditions. The Chinese government’s “Dynamic COVID-zero” strategy has withstood severe challenges and is now tackling the Omicron variant effectively [5]. Admittedly, at this stage, totally defeating the virus seems problematic because of substantial economic losses and public appeals for reopening. Therefore, some countries are no longer at war with the coronavirus; instead, they are working out how best to coexist with its presence [6]. However, whatever strategy is implemented, regulating public behaviors is a key element in response to the COVID-19 pandemic. Published research indicates that strict preventative measures and good behavioral practice would greatly influence morbidity and mortality rates, which would help control disease spread [7].

The COVID-19 outbreak, as a grave global public health emergency, poses new challenges to the worldwide risk emergency management system. Consequently, identifying the key factors affecting public behavior has great practical significance in constructing high-level emergency risk management systems. Research shows that many factors influence public behavior during the pandemic, whether external factors such as risk communication [8], trust level [9], etc., or internal psychological factors including risk perception [10, 11], negative emotions [12], etc. However, government crisis interventions based on identification of single influencing factors have been severely challenged by the emergence over recent years of complex social problems such as public health emergencies. A single influencing factor intervention often directly brings about linkage changes of other multiple factors, the so-called “a slight move in one part may affect the situation as a whole” effect. Therefore, policymakers must effectively identify configuration conditions that affect public behavior and their synergistic effects, selecting adaptive crisis intervention strategies in combination with the specific conditions of different governments.

In the context of complex social studies, it is impossible to design, manage and control all factors, so we focus on the key factors that must be present–the necessary conditions [13]. To this end, based on relevant literature reviews and practical observations, this study came up with relevant condition variables for two main reasons: first, the conditions influencing outcomes should be in line with theoretical logic. Second, the variables should exist objectively and can be intervened. In early research, we constructed a public behavioral coping strategies model during the COVID-19 pandemic. We used structural equation modeling to conduct preliminary demonstrations [14]. However, we have not conducted an in-depth discussion of the reasons for differences in public behavior coping strategies. Specifically, the paper lacks an analysis of core conditions and configurations of various influencing factors that lead to different public behavior coping strategies and cannot systematically reveal the complex operating mechanism of multi-element interactions in public behavior during the COVID-19 pandemic, which will undoubtedly restrict further development of the public behavior framework theory.

A “configuration perspective” is widely used in complex social crisis interventions to understand the causal complexity behind outcomes. This perspective indicates that influencing factors are interdependent, and expected results can be achieved through differentiated permutation and combination with various factors. This study further adopted the “configuration perspective” to empirically explore public behavior influencing factors and improvement paths. Specifically, we aimed to answer the following research questions: What configuration of conditions influences public behavior? Which conditions play a more critical role? What kind of matching and substitution relationship exists between them?

Accordingly, based on the proposed framework, we attempt to reveal differences in the conditional configuration and mechanism of public behavior through a fuzzy set qualitative comparative analysis (fsQCA). By discussing the synergistic effect of multiple conditions in the framework and the complex interactive nature of various conditions driving public behavior, our study could make up for the deficiencies of existing research in explaining such issues as “How to promote the public’s protective behavior or reduce the public’s excessive behavior?” and finally provide new evidence and ideas for the government to improve the emergency management system.

## Literature review and analysis framework

### Public behavioral coping strategies model framework during the COVID-19 pandemic

More risk studies from social sciences have realized that the intervention of public behavior on risk is crucial [15]. Several behavioral theories have been established and extended. The *theory of planned behavior* (*TPB*) is an explanatory model widely applied in studies on behavioral intention [16]. Public behavior theory based on *TPB* revealed that attitude, perceived behavior control (PBC), and people’s knowledge significantly affect their behavior in reducing urban air pollution [17]. Other similar behavioral models, like the recycling behavior model based on TPB, were also reported [18]. Besides, people generate a *cognition-affect-coping model* when facing threats and pressure; that is, an individual’s cognition and judgment of risk stimuli produce a corresponding effect and then influence the individual’s response behavior [19]. A serial mediation model based on *cognition-affect theory* showed that scarcity aggravates panic buying, and this aggravation effect is serially mediated by perceived control and panic [20]. These findings provide essential enlightenment for guiding rational public behavior and managing public opinion during emergencies. Besides, an exploratory theoretical model of four public behaviors based on the combinations of the public-personal domains and mitigation-adaptation actions was suggested against the risk of particulate matter (PM, a small air pollutant) by focusing on the roles of risk perception, communication, and attribution factors [21]. These results provide some enlightenment effect on selecting public behavioral factors and classifying behavioral coping strategies in our research.

However, the previous theories and models (TPB, cognition-affect theory) lack specificity in the context of COVID-19 crisis, and the specific public behavior theories were rarely reported. Furthermore, traditional statistical methods, such as principal component analysis, linear regression model, and path analysis [17, 18, 20, 21], only clarify the quantitative correlation between the dependent and independent variables and cannot profoundly explain its further logical causality, which limits the theory’s explanatory power.

When facing the severity of the COVID-19 pandemic, the public can respond in different ways, varying from protective coping behavior (PCB) to excessive coping behavior (ECB).

On the one hand, people can take preventive actions to reduce their risk. A vaccination strategy is considered the best option for COVID-19 prevention, as the vaccine can protect public health and reduce transmission of the virus. Still, skepticism, hesitancy, and resistance remain [22–24]. Research shows that individual vaccination decisions are related to personal characteristics and rooted in their public health and home state’s political and economic contexts [25]. Safe and effective vaccines are undoubtedly groundbreaking. Nevertheless, the resurgence of the COVID-19 crisis occurred in many countries in the latter half of 2021 due to waning immunity from vaccination after the second dose [26].

Consequently, the public was recommended to continue wearing masks, washing their hands, ensuring good ventilation indoors, physically distancing, and avoiding crowds in the foreseeable future [27]. Social distancing is very effective in blocking short-distance infections. Mathematical modeling demonstrates that social distancing and public behavior changes had curbed the spread of COVID-19 [15]. Public behavior was deeply affected by local government regulations rather than the global pandemic situation [28]; there were noticeable regional differences in intent to follow key public health recommendations such as “stay home and keep social distancing” [29].

On the other hand, the public may also respond with excessive actions. Panic buying is a common phenomenon during public emergencies and has substantially undesirable social impacts [20]. The media must consider the effect of their messaging on public behavior, as even imagined food shortages can instigate excessive actions such as stockpiling and panic buying behavior, as observed during the COVID-19 pandemic [30].

As a critical link in the emergency response process, risk communication (RC) transmits real-time information, advice, and opinions between experts and people facing threats to their health, economic, or social well-being [31], which can profoundly affect public behaviors during the pandemic. An online randomized controlled trial demonstrated the importance of effective RC in reducing undesired public behavior during non-conventional terrorism crises [32]. The government could use relevant media as a crisis and risk communication strategy to intervene in pandemic-related public behavior [8]. Research suggests that vaccine efforts might need to go beyond communication campaigns correcting misinformation about COVID-19 vaccines and should focus on re-establishing public trust in government agencies [33]. Trust in science may positively affect individuals’ social distancing behavior by decreasing perceived media exaggeration about COVID-19 [34]. Trust is a critical factor that encourages people to comply with public health regulations. One online survey showed that higher trust in governmental organizations was related to greater compliance in adopting protective behaviors during the COVID-19 crisis [9].

The studies above indicate that the critical external factors, RC and degree of trust (DT), can profoundly affect pandemic-related public behavior. Their regulation by risk management departments may be effective in pandemic prevention and control. Furthermore, we noted that today, the public’s psychological factors are closely related to external factors and are becoming more critical in better targeted psychological pandemic-crisis interventions.

Public risk perception (RP) is defined as the subjective judgment people make about the characteristics and severity of risks [35], which is an essential consideration in public health emergencies and risk management decision-making [36]. A qualitative study revealed that people influenced by information and advice campaigns perceive a risk that has shaped their protective behavior [10]. Moreover, there is a dynamic relationship between RP and pandemic-related behavior [11]. Therefore, timely monitoring and regulating public RP can help the government predict public behavior and manage risk effectively. In addition to RP’s impact on behavior, emotion plays a central role. Higher RP concerning COVID-19 is notably associated with less favorable or more negative emotions (NE) [12]. Events during the public health crisis (like lockdown) increase the likelihood of public NE (worry, fear, and anxiety), which in turn prompt behaviors including excessive avoidance and blind obedience [37]. Hence, it is crucial to grasp the potential psychological effects of COVID-19 immediately.

**Fig. 1:**
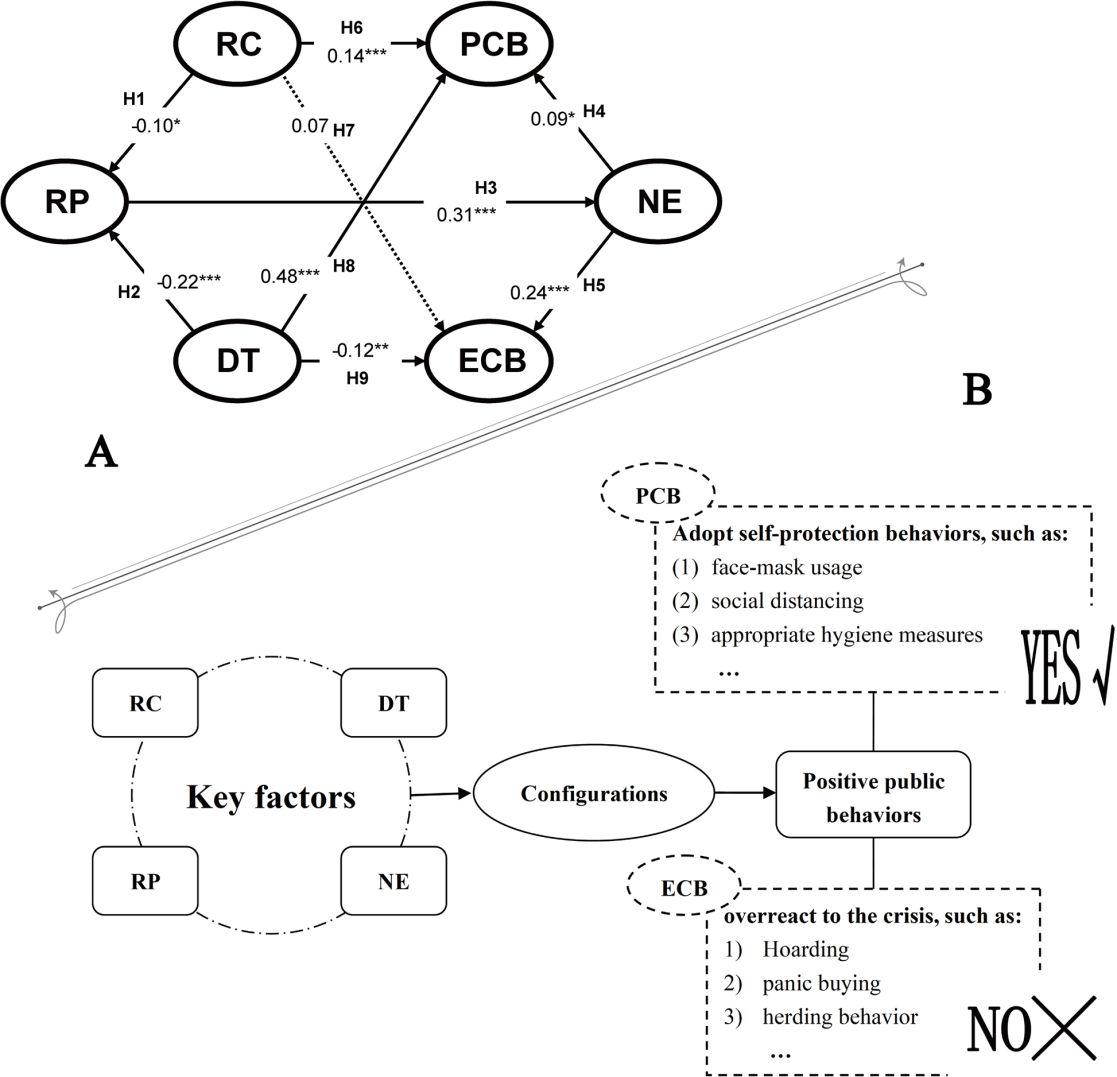
Theoretical and analytical framework. **A** Public behavioral coping strategies model [14]; **B** Analysis framework. RC, Risk communication; DT, degree of trust; RP, risk perception; NE, negative emotions; PCB, protective coping behavior; ECB, excessive coping behavior

In conclusion, public behavior is shaped by key external (RC and DT) and internal (RP and NE) influencing factors, and we constructed the behavioral framework hypothesis. Our previous study [14] verified the theory by path analysis (Fig. 1A). However, the asymmetry of causality in social problems and the correlation of multiple causes limit further interpretation of the results. From the configuration perspective, the influences of external factors and internal psychological conditions on public behavior response are not independent; still, they play a synergistic role through linkage and matching (Fig. 1B). Specifically, concurrent synergistic effects among multiple conditions may include mutual reinforcement through adaptation or cancellation through substitution. Therefore, from a configuration perspective, the study empirically explores how external–internal conditions can affect public behavior through mutual matching (adaptation/substitution).

### The fuzzy set qualitative comparative analysis

Herein, we attempt to analyze the multiple driving mechanisms behind public behavior during the COVID-19 pandemic based on a configuration perspective, so we proposed using fsQCA to carry out an empirical test.

Ragin proposed the qualitative comparative analysis (QCA) method in the 1980s [38]. In QCA, researchers can determine the logical relationship between matching configurations of different conditions and outcomes through cross-case comparison, that is, “Which configurations of condition variables can lead to the appearance or disappearance of outcomes?” thereby further identifying the synergistic effects of multiple conditional variables under the premise of acknowledging causal complexity.

In delineating the social sciences approaches, it is conventional to distinguish between quantitative, variable-oriented analysis and qualitative, case-oriented analysis [38, 39]. The fundamental objective of variable-oriented research is the production of descriptive or explanatory inferences by hypothesis testing [39]. In contrast, case-oriented qualitative research is more valuable when implemented to spotlight each case’s distinctiveness and facilitate theory development [39]. By embracing both quantitative and qualitative methods’ aspects, comparative methods can circumvent some of both approaches’ limitations [38, 39]. Like case-oriented methods, comparative methods maintain cases’ integrity; like variable-oriented methods, comparative methods examine relationships’ patterns among variables. So, comparative methods, described as a ‘bridge’ between qualitative, case-oriented research and quantitative, variable-oriented research [40], could be applied for both hypothesis testing and theory development [38]. Unlike variable-oriented causal research methods, such as regression analysis or path analysis, that produce precise predictions of the likely effect of one variable upon another [41], comparative methods see the social world in terms of sets and set-theoretic relations [42], which emphasizes the search for highly consistent relationships linking combinations of causes to outcomes [40].

Rooted in set theory, QCA uses set algebra – also known as Boolean algebra – to analyze causal configurations [42, 43]. QCA has many advantages: on the one hand, researchers can identify conditional configurations with equivalent outcomes, which can help people understand the differential driving mechanisms that lead to outcomes in different scenarios, and further discuss the adaptation and substitution relationship between conditions. On the other hand, researchers can further compare the configuration of conditions that lead to the emergence and disappearance of outcomes and broaden their theoretical interpretation of specific research questions. Under the logical premise of causal asymmetry, the conditions that lead to the emergence of the outcomes may not be the same as those that lead to the disappearance of the outcomes. QCA includes three basic categories [13, 43]: a clear set qualitative comparative analysis (csQCA), fsQCA, and multi-valued set qualitative comparative analysis (mvQCA). Compared with the characteristics of csQCA and mvQCA, which are only suitable for dealing with category problems, fsQCA can further deal with the problem of degree change or partial membership [44].

Accordingly, based on the above theoretical model framework, our study attempts to analyze the multiple driving mechanisms behind public behavioral coping strategies during the COVID-19 pandemic from a configuration perspective. Therefore, fsQCA is proposed to conduct empirical tests.

## Methods

### Questionnaire and variable measurement

Data were elicited using a self-designed questionnaire in line with the relevant literature. A total of 735 valid cases were obtained by conducting an online survey in China from April to July, 2020. The procedure of data collection and variable measurement are explained in the published literature [14].

Herein, we explore public behavioral responses, including two outcome variables, PCB and ECB. Four condition variables were adopted: RC, DT, RP, and NE. See Supplementary Table S1.

### Calibration of fsQCA conditions

In fsQCA, calibrating refers to assigning set membership to cases [45]. Specifically, researchers need to calibrate variables into sets based on existing theoretical knowledge and case context. The calibrated ensemble membership will be between 0 and 1.

To select the anchor points, we refer to the relevant literature [46] and the actual situation of the data to make the final determination. Specifically, variable including PCB, RC, DT, RP, and NE was assessed using a 5-point Likert scale (Table 1). Anchor points of maximum uncertainty were adopted by mean of variables, while full membership was by mean + standard deviation (SD), and non-membership by mean – SD. ECB was assessed using three multiple-choice questions; one point was assigned for each type of behavior, ranging from 0–4 points for each item. Hence, 0 represents non-membership, 1 maximum uncertainty, and 4 full membership.

**Table 1:** Calibration anchor points and descriptive Statistics of variables

**Variable**	**Anchor points**			**Descriptive analysis**			
	**Full membership**	**Maximum uncertainty**	**Non-membership**	**Mean**	**SD**	**Min**	**Max**
PCB	5.000	4.320	3.610	4.320	0.710	1	5
ECB	4.000	1.000	0.000	1.057	0.955	0	4
RC	4.804	4.040	3.276	4.040	0.764	1	5
DT	4.970	4.246	3.522	4.246	0.724	1	5
RP	2.967	2.232	1.497	2.232	0.735	1	5
NE	3.641	2.818	1.995	2.818	0.823	1	5

*RC* Risk communication, *DT* Degree of trust, *RP* Risk perception, *NE* Negative emotions, *PCB* Protective coping behavior, *ECB* Excessive coping behavior, *SD* Standard deviation, *Min* the minimum value, *Max* the maximum value

### QCA and necessary condition analysis

Necessary and sufficient causality are two emerging explanations of causality [47, 48], in which necessary conditional causality means that the outcome will not occur if a specific antecedent does not exist [45]. The fsQCA analysis can effectively identify the necessary conditions, states “whether a condition is necessary or unnecessary for an outcome,” but does not quantitatively reflect the degree “to what extent a condition is necessary for an outcome” [13, 49]. Therefore, to better analyze necessary and sufficient causality, we adopted a new method of Necessary Condition Analysis (NCA), which is a data analysis approach that estimates the necessity effect size of a condition for an outcome [13, 50]. Especially for fuzzy sets data, if the value is not just “yes = 1” or “no = 0,” but also membership, the approach combination of NCA and fsQCA is more valuable. Therefore, this study applied fsQCA using the fsqca3.0 software (University of California, Irvine, CA, USA) for empirical analyses, and R software (version 3.5) was used for NCA; *p* values ≤ 0.05 were statistically significant.

## Results

### Analysis of necessary conditions

The necessity and sufficiency of subset relations are generally evaluated through the set-theoretic measures of consistency and coverage [45]. Consistency reflects the degree of membership of a condition to a configuration [48]. The coverage of a configuration refers to the percentage of cases that can be explained by that configuration [48].

We first examine the “Necessity” of each condition individually. Single-factor necessity analysis showed that none of the consistency of the conditions (or the absence of the conditions marked by “ ~ ”) exceeded the cut-off value of 0.9 [44, 45], indicating that no single condition by itself was a necessary condition of PCB or ECB (or the absence of PCB or ECB).

The coverage of most conditions for outcome was higher than 0.5 [45], meaning that each condition has enough explanatory strength on the outcome variables. Among them, the coverage of DT for PCB was larger than 0.75 (= 0.774), suggesting that DT explained a moderate number of cases in which the public adopt protective coping behavior (PCB). See Table 2.

**Table 2:** Analysis of necessary conditions

Conditions	Outcome							
	**PCB**		** ~ PCB**		**ECB**		** ~ ECB**	
	**Consistency**	**Coverage**	**Consistency**	**Coverage**	**Consistency**	**Coverage**	**Consistency**	**Coverage**
RC	0.649	0.715	0.509	0.439	0.638	0.521	0.614	0.705
~ RC	0.491	0.561	0.670	0.599	0.639	0.541	0.583	0.694
DT	0.748	0.774	0.475	0.385	0.631	0.485	0.668	0.721
~ DT	0.407	0.498	0.722	0.692	0.637	0.577	0.523	0.666
RP	0.484	0.596	0.625	0.603	0.636	0.580	0.552	0.708
~ RP	0.677	0.698	0.581	0.469	0.680	0.520	0.673	0.722
NE	0.564	0.627	0.591	0.515	0.686	0.565	0.555	0.643
~ NE	0.564	0.638	0.572	0.507	0.567	0.476	0.624	0.736

*RC* Risk communication, *DT* Degree of trust, *RP* Risk perception, *NE* Negative emotions, *PCB* Protective coping behavior, *ECB* Excessive coping behavior

~ represent the absence of the condition or outcome

Overall, this result shows the complexity of pandemic-related public behavior influencing factors. Multiple conditions were linked and matched to each other to affect public behavior responses jointly. That is, risk management based on public behavior should be the concurrent synergistic effect of multiple conditions under the four aspects of RC, DT, RP, and NE.

NCA explores the minimum necessary conditions required to produce a particular outcome by analyzing the effect sizes of the necessary conditions [49]. According to the relevant literature, the necessary conditions of outcome in the NCA method need to meet two requirements: (1) the effect size is at least 0.1 [50], and (2) the Monte Carlo simulations of permutation tests show that the effect size is statistically significant [13].

Herein, we report the NCA results (Table 3), including accuracy (%), ceiling zone, scope, effect size, and *p*-value, from two different estimation methods [49]. The first method is the non-parametric Ceiling Envelopment with Free Disposal Hull (CE-FDH), which is a piecewise linear line. The second is the parametric Ceiling Regression with Free Disposal Hull (CR-FDH). This method smoothens the piecewise linear lines using a straight line. Unlike the CE-FDH, CE-FDH is 100% accurate in drawing the demarcation between observations above and below the ceiling line. The NE condition for ECB is statistically significant (*p* < 0.05), but the effect size is too small to be considered a necessary condition for high-level ECB. The results for other conditions were not statistically significant (*p* > 0.05), showing that they are neither necessary for high-level PCB nor ECB. Furthermore, we report the bottleneck analysis results. The bottleneck level represents the minimum necessary conditions required to produce a specific outcome. As seen in the bottleneck level table (Table 4), to reach a 90%-level outcome of ECB, conditions of 2.0%-level RC or 5.0%-level NE are required. There is no bottleneck level of other conditions for public behavior.

**Table 3:** The necessity analysis result based on NCA

**Conditions**	**Method**	**PCB**					**ECB**				
		**Accuracy (%)**	**Ceiling zone**	**Scope**	**Effect size**	***P*-value**^a^	**Accuracy (%)**	**Ceiling zone**	**Scope**	**Effect size**	***P*-value**^a^
RC	CE-FDH	100	0.000	0.930	0.000	1.000	100	0.003	0.882	0.003	0.310
	CR-FDH	100	0.000	0.930	0.000	1.000	100	0.001	0.882	0.001	0.310
DT	CE-FDH	100	0.000	0.910	0.000	1.000	100	0.000	0.864	0.000	0.658
	CR-FDH	100	0.000	0.910	0.000	1.000	100	0.000	0.864	0.000	0.658
RP	CE-FDH	100	0.000	0.940	0.000	1.000	100	0.000	0.891	0.000	1.000
	CR-FDH	100	0.000	0.940	0.000	1.000	100	0.000	0.891	0.000	1.000
NE	CE-FDH	100	0.000	0.950	0.000	1.000	100	0.006	0.900	0.007	0.018^*^
	CR-FDH	100	0.000	0.950	0.000	1.000	100	0.003	0.900	0.004	0.034^*^

*RC* Risk communication, *DT* Degree of trust, *RP* Risk perception, *NE* Negative emotions, *PCB* Protective coping behavior, *ECB* Excessive coping behavior, *CE-FDH* the non-parametric Ceiling Envelopment with Free Disposal Hull, *CR-FDH*, the parametric Ceiling Regression with Free Disposal Hull

^a^ Permutation test in NCA with 10,000 repetitions

^*^ *p* < 0.05

**Table 4:** Bottleneck analysis result (%)

CE-FDH ^a^	PCB				ECB			
	**RC**	**DT**	**RP**	**NE**	**RC**	**DT**	**RP**	**NE**
0	NN	NN	NN	NN	NN	NN	NN	NN
10	NN	NN	NN	NN	NN	NN	NN	NN
20	NN	NN	NN	NN	NN	NN	NN	NN
30	NN	NN	NN	NN	NN	NN	NN	NN
40	NN	NN	NN	NN	NN	NN	NN	NN
50	NN	NN	NN	NN	NN	NN	NN	NN
60	NN	NN	NN	NN	NN	NN	NN	NN
70	NN	NN	NN	NN	NN	NN	NN	NN
80	NN	NN	NN	NN	NN	NN	NN	NN
90	NN	NN	NN	NN	2.0	NN	NN	5.0
100	NN	NN	NN	NN	2.0	1.0	NN	5.0

*RC* Risk communication, *DT* Degree of trust, *RP* Risk perception, *NE* Negative emotions, *PCB* Protective coping behavior, *ECB* Excessive coping behavior

^a^ CE-FDH (the non-parametric Ceiling Envelopment with Free Disposal Hull) method was adopted, and NN is “not necessary.”

The results of the NCA method are consistent with the QCA results. There is no single necessary condition for producing high levels of PCB and ECB.

### Analysis of sufficient conditions

We further carried out sufficient conditional analysis to obtain the conditional configuration with the most significant explanatory power for each outcome. We committed to finding configuration for positive pandemic-related public behaviors. Therefore, the conditional configuration for encouraging people to adopt more protective behavior (the outcome PCB) and less excessive action (the outcome ~ ECB) were empirically tested.

Relevant literature recommended that raw consistency thresholds of sufficient conditions analysis were not less than 0.75 [45], which generally were set at 0.75 [45], 0.8 [42], or natural truncation point [51]. Combined with the data characteristics, this study sets the consistency thresholds of configuration analysis of PCB and ~ ECB to 0.8 (cutoff = 0.812) and 0.9 (cutoff = 0.909), respectively. In fuzzy set analysis, it is also essential to consider PRI consistency (proportional reduction in inconsistency) to avoid simultaneous subset relations of configurations in both the outcome and its absence. PRI consistency should be high and ideally not too far from raw consistency. Herein, a PRI consistency threshold of 0.6 was recommended [52], and the case frequency threshold was set to 10, about 1.5% of the sample size [42]. Truth Table Analysis can be seen in Supplementary Table S2 and Table S3.

First, the results show two driving paths (S1 and S2) to encourage people to adopt protective behavior. The overall solution consistency is 0.800, indicating that 80.0% of cases that meet these two conditional configurations adopt high PCB. The overall solution coverage is 0.686, showing that these two types of conditional configurations explain 68.6% of the cases that adopted high-level PCB. See Table 5

**Table 5:**
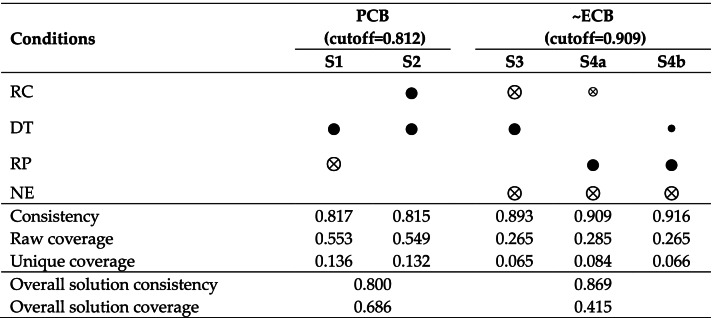
A configuration analysis of the public adopting positive behavior

= the core condition exists,  = the core condition is absent,  = the auxiliary condition exists, and  = the auxiliary condition is absent. A blank space indicates that the condition can exist or be absent

*RC* Risk communication, *DT* Degree of trust, *RP* Risk perception, *NE* Negative emotions, *PCB* Protective coping behavior, *ECB* Excessive coping behavior

~ represent the absence of the condition or outcome. S1, S2, S3, S4a, and S4b represent different configurations

.

From a practical viewpoint, risk management departments should focus on promoting RC and reducing RP while adopting a high-level DT strategy, which is conducive to the public taking protective behaviors. These three factors are core conditions, among which high-level DT is the core condition in each solution, and high-level RC and low-level RP are alternative conditions for each other.

Second, there are three driving paths (S3, S4a, and S4b) to inhibit ECB. The overall solution consistency is 0.869, revealing that 86.9% of cases that match these three conditional configurations engage in low-level ECB. The overall solution coverage is 0.415, demonstrating that these two conditional configurations explain 41.5% of the cases that adopted low-level ECB. See Table 5.

Lower NE is considered a core condition to help restrain the public’s ECB in each solution path. Related departments should pay special attention to regulating public emotions in risk management based on public behavior. Keeping public sentiment stable and avoiding accumulation of NE is crucial. In paths S4a and S4b, high-level DT and low-level RC are alternative conditions.

### Robustness test

Researchers usually judge whether the results are robust by adjusting the calibration of conditions, the threshold of raw consistency and case frequency, or randomly deleting cases. Considering that we have a large enough sample size, we conducted a robustness test on the antecedent configurations of PCB and ~ ECB by randomly deleting half the cases (*n* = 367). As shown in Supplementary Table S4 and Table S5, our results are sufficiently robust.

## Discussion and conclusions

In the foreseeable future, society must combat the COVID-19 pandemic. Government departments should conduct effective risk management based on public behavior. Encouraging the public to take positive responses is a crucial measure that risk management departments need to consider. Specifically, relevant departments need to encourage the public to take protective behaviors as much as possible, such as wearing masks, practicing social distancing, and vaccination. Meanwhile, society could take further measures to discourage people from taking excessive behaviors, such as hoarding [53], and herding behavior (a bandwagon effect led by rumors) [54].

The pandemic-related public behavior response was affected by various factors. Our previous research explored vital external and internal factors such as RC, DT, RP, and NE that significantly influence public response [14], which quantitatively revealed the above individual factors’ effects on public behavior. However, considering the complexity of social issues, which are often influenced by multiple factors and have complex interactive mechanisms, bias is unavoidable if only relying on analysis to study the individual effects of various factors on public behavior. Moreover, existing studies have not thoroughly explored core or auxiliary conditions that affect public behavior. Therefore, we adopted a “configuration perspective” to further understand the causal complexity behind public coping behavior when facing a crisis like such COVID-19 pandemic. Herein, we collected 735 valid cases through online questionnaires and used fsQCA to conduct conditional configuration analysis, empirically testing the public behavioral coping framework proposed earlier and further exploring the driving path of key factors affecting public protective or excessive behavior during the COVID-19 pandemic.

Our study found that: (1) critical factors including RC, DT, RP, and NE alone could not constitute a necessary condition for public protective behavior or excessive behavior. (2) Two configurations constitute the driving path for promoting public protective behavior. Trust is considered the core condition in both paths, which suggests that compared with other conditions, enhancing the public’s trust is a crucial strategy for government. The substitution relationship of RC, DT, and RP indicated that combining high-level trust with high-level RC or low-level RP could improve public protective behavior in a “similar outcome” way through substitution. (3) The three configurations constitute the driving path to restraining excessive public behaviors. The most important thing is that low-level NE can effectively suppress excessive behavior regardless of the path. (4) Interestingly, regarding the impact of RP on public behavior, we did not observe similar previous findings that high levels of RP can encourage the public to take protective and aggressive behaviors, which suggested the mechanism of individual conditions on public behavior is different from configuration conditions. RP is a complex measurement dimension, but we merged them, which undoubtedly lost certain information in the procedure of the QCA test, which might further result in inconsistent outcomes. (5) An effective behavioral risk management policy should encourage the public to adopt protective behaviors and reduce excessive behavior.

To sum up, the configurations of various influencing factors reveal the complexity of public behavioral risk management. For behavioral risk management, governments should focus on adapting to multiple conditions according to their situations and, under the “overall perspective,” formulate policies based on local conditions and further form a differentiated risk management path. Practically speaking, for the government, taking adequate measures to increase public trust and reduce negative public emotions is the core path of risk management to enhance positive public behavior.

We believe that the discussion in this paper will further elaborate on the public behavior framework model proposed earlier. Specifically, based on the quantitative analysis model, we qualitatively compared the different configurations from the perspective of configuration analysis, empirically explored the concurrent synergistic effect and linkage matching mode of multiple conditions in promoting public behavior, and further expanded the application of the public behavior framework in explaining “causal complexity,” and solved the current dilemma faced by the theory to a certain extent. However, this study also has some limitations. First, this study aimed to analyze the complex interaction mechanism of different key factors and how to affect public behavior through case comparison. The fsQCA could allow researchers to conduct a more in-depth within-case analysis. However, it does not answer research questions such as “why” as satisfactorily as a deep longitudinal case study. Therefore, future research needs to combine observational research, in-depth interviews, and other methods to further explore the mechanism deeply between influencing factors and public behavior. Second, this study only analyzed data in the early pandemic because of availability. Nevertheless, the factors impacting public behavior may not be stable, and the key factors affecting public behavior at different stages are also various. Specifically, we did not compare the case data in the early, middle, and late stages, limiting the explanatory power of the research conclusions in the time dimension.

## Supplementary Information

**Additional file 1: Table S1.** Measurement of variables. **Table S2.** Truth Table Analysis of PCB.** Table S3.** Truth Table Analysis of ~ECB. **Table S4.** Robustness Test of PCB. **Table S5.** Robustness Test of ~ECB.

## Data Availability

The data-sets analyzed during the current study are available from the corresponding author on reasonable request.
