# Supplementary material for: Configurations for positive public behaviors in response to the COVID-19 pandemic: a fuzzy set qualitative comparative analysis
Source: BMC Public Health. 2022 Sep 6;22:1692. doi: 10.1186/s12889-022-14097-6 (PMC9449292; doi:10.1186/s12889-022-14097-6)
Supplement: Supplementary file 1 — Additional file 1: Table S1. Measurement of variables. Table S2. Truth Table Analysis of PCB. Table S3. Truth Table Analysis of ~ECB. Table S4. Robustness Test of PCB. Table S5. Robustness Test of ~ECB. [file 12889_2022_14097_MOESM1_ESM.docx]

Table S1 Measurement of variables

| **Variable** | **Description** | **Measurement** |
| --- | --- | --- |
| PCB | - | PCB was measured using a 5-point Likert scale (ranging from 1 “*never*” to 5 “*always*”) in relation to the following questions: “Did you reduce contact with others during the pandemic?”; “Did you always wear a mask when you went out during the pandemic?”; “Did you try to avoid going to crowded places for activities during the pandemic?”; “Did you increase the amount of handwashing during the pandemic?”; “Did you open windows more often for ventilation during the pandemic?”; “Did you reduce the number of dinner parties held during the pandemic?”; “Did you increase exercise during the pandemic?”; “Were you more proactive in paying attention to and seeking health information during the pandemic?”; “Did you remind your family or friends to take measures to prevent and treat infection due to COVID-19 during the pandemic?”; and “Did you try to avoid contact with wild animals during the epidemic?” |
| ECB | - | ECB was assessed using three multiple choice questions, as follows: “Did you panic buy during the pandemic? If yes, please indicate whether any of the following items were involved: personal protective equipment, disinfectant, antiviral drugs, other directly pandemic-related purchases, or none of these”; “Did you hoard during the pandemic? If yes, please indicate whether any of the following items were involved: personal protective equipment, disinfectant, antiviral drugs, other directly pandemic-related purchases, or none of these”; “Were you influenced by a bandwagon effect led by rumor (herding behavior)? If yes, please indicate whether any of the following was involved: taking Shuanghuanglian (a drug with no preventive effect) to prevent COVID-19, sterilizing with white vinegar, taking other antiviral drugs while not been infected, other similar behavior, or none of these.” One point was assigned for each type of behavior, with points ranging from 0-4 points for each item. |
| RC | RC, as a key link in the entire process of emergency response, is defined as the exchange of real-time information, advice, and opinions between experts and people facing threats to their health, economic, or social well-being. | A 5-point Likert scale, where 1 corresponded to “*totally disagree*” and 5 to “*totally agree*” for the following three questions: “Do you think media reports affected your judgment of the COVID-19 pandemic?”; “Do you believe media reports on COVID-19 are true?”; and “Do you believe that government departments are timely and transparent in the release of pandemic information?” |
| DT | Trust involves an overall positive expectation concerning the worthiness of words, promises, and statements of either another person (interpersonal trust) or an institution (social trust). COVID-19-related trust could be effectively measured in terms of beliefs related to “competency – having technical proficiency.” | The degree of trust (DT) was measured as the perceived or expected trustworthiness of others (e.g., government, medical workers), which was assessed using a 5-point Likert scale where 1 corresponded to “*very distrusting*” and 5 to “*very trusting*” for the following 4 questions: “Can the pandemic be effectively controlled?”; “What is your DT in the pandemic control ability of medical workers?”; “What is your DT in your self-protection capabilities?”; and “What is your DT in the government’s ability to prevent and control the pandemic?” |
| RP | Slovic claimed that people’s RP of crisis events can be measured from the two dimensions of familiarity and controllability. | In this study, we chose controllability as the RP measurement index, with five aspects of risk events investigated, namely, etiology, transmission, cure, preventive measures, and prognosis. RP was assessed using a 5-point Likert scale for the following five questions: “In terms of the controllability of the COVID-19 pandemic, what is your RP concerning its etiology, transmission, cure, preventive measures, and prognosis? (Responses ranging from: 1 “*totally controllable*” to 5 “*totally uncontrollable*”). |
| NE | - | NE were assessed using a 5-point Likert scale (ranging from 1 “*never*” to 5 “*always*”) in relation to the following questions: “What was your worry, fear, and anxiety frequency during the COVID-19 pandemic?” |

Note: RC, Risk communication; DT, degree of trust; RP, risk perception; NE, negative emotions; PCB, protective coping behavior; ECB, excessive coping behavior.

**Table S2** Truth Table Analysis of PCB

| **RC** | **DT** | **RP** | **NE** | **number** | **PCB** | **raw consistency** | **PRI consistency** | **SYM consistency** |
| --- | --- | --- | --- | --- | --- | --- | --- | --- |
| 1 | 1 | 0 | 1 | 62 | 1 | 0.8591 | 0.7645 | 0.7759 |
| 1 | 1 | 0 | 0 | 79 | 1 | 0.8526 | 0.7700 | 0.7881 |
| 0 | 1 | 0 | 1 | 52 | 1 | 0.8441 | 0.6999 | 0.7041 |
| 1 | 1 | 1 | 1 | 35 | 1 | 0.8402 | 0.7168 | 0.7365 |
| 1 | 1 | 1 | 0 | 15 | 1 | 0.8228 | 0.6553 | 0.6618 |
| 0 | 1 | 0 | 0 | 65 | 1 | 0.8118 | 0.6588 | 0.6608 |
| 0 | 1 | 1 | 1 | 25 | 0 | 0.7953 | 0.5691 | 0.5816 |
| 0 | 1 | 1 | 0 | 15 | 0 | 0.7758 | 0.5384 | 0.5390 |
| 1 | 0 | 0 | 1 | 31 | 0 | 0.7191 | 0.4636 | 0.4690 |
| 1 | 0 | 0 | 0 | 15 | 0 | 0.7121 | 0.4293 | 0.4325 |
| 1 | 0 | 1 | 1 | 10 | 0 | 0.7045 | 0.3899 | 0.3903 |
| 0 | 0 | 0 | 1 | 74 | 0 | 0.6941 | 0.4322 | 0.4391 |
| 0 | 0 | 0 | 0 | 68 | 0 | 0.6393 | 0.3574 | 0.3606 |
| 0 | 0 | 1 | 0 | 31 | 0 | 0.6297 | 0.3250 | 0.3284 |
| 0 | 0 | 1 | 1 | 90 | 0 | 0.5764 | 0.3031 | 0.3078 |

Note: RC, Risk communication; DT, degree of trust; RP, risk perception; NE, negative emotions; PCB, protective coping behavior.

**Table S3** Truth Table Analysis of ~ECB

| **RC** | **DT** | **RP** | **NE** | **number** | **~ECB** | **raw consistency** | **PRI consistency** | **SYM consistency** |
| --- | --- | --- | --- | --- | --- | --- | --- | --- |
| 0 | 1 | 1 | 0 | 15 | 1 | 0.9479 | 0.8332 | 0.8574 |
| 1 | 1 | 1 | 0 | 15 | 1 | 0.9293 | 0.7936 | 0.8474 |
| 0 | 0 | 1 | 0 | 31 | 1 | 0.9213 | 0.7691 | 0.8168 |
| 0 | 1 | 0 | 0 | 65 | 1 | 0.9093 | 0.7761 | 0.8070 |
| 1 | 0 | 1 | 1 | 10 | 0 | 0.8999 | 0.6428 | 0.6588 |
| 0 | 1 | 1 | 1 | 25 | 0 | 0.8993 | 0.6879 | 0.7130 |
| 0 | 1 | 0 | 1 | 52 | 0 | 0.8887 | 0.7114 | 0.7426 |
| 1 | 0 | 0 | 0 | 15 | 0 | 0.8882 | 0.6678 | 0.6891 |
| 1 | 1 | 0 | 0 | 79 | 0 | 0.8810 | 0.7583 | 0.8450 |
| 0 | 0 | 0 | 0 | 68 | 0 | 0.8742 | 0.6797 | 0.7022 |
| 0 | 0 | 0 | 1 | 74 | 0 | 0.8559 | 0.6152 | 0.6567 |
| 1 | 1 | 0 | 1 | 62 | 0 | 0.8483 | 0.6582 | 0.7042 |
| 1 | 0 | 0 | 1 | 31 | 0 | 0.8429 | 0.5668 | 0.5944 |
| 1 | 1 | 1 | 1 | 35 | 0 | 0.8303 | 0.5602 | 0.6225 |
| 0 | 0 | 1 | 1 | 90 | 0 | 0.8092 | 0.5275 | 0.5757 |

Note: RC, Risk communication; DT, degree of trust; RP, risk perception; NE, negative emotions; ECB, excessive coping behavior. ~ represent the absence of the condition or outcome.

**Table S4** Robustness Test of PCB

| **Conditions** | **PCB** | | | | |
| --- | --- | --- | --- | --- | --- |
|  | **(n=735, cutoff=0.812)** | |  | **(n=367, cutoff=0.803)** | |
|  | **S1** | **S2** |  | **S1** | **S2** |
| RC |  | ● |  | ● |  |
| DT | ● | ● |  | ● | ● |
| RP | ⊗ |  |  |  | ⊗ |
| NE |  |  |  |  | ● |
| Consistency | 0.817 | 0.815 |  | 0.796 | 0.830 |
| Raw coverage | 0.553 | 0.549 |  | 0.557 | 0.330 |
| Unique coverage | 0.136 | 0.132 |  | 0.302 | 0.075 |
| Overall solution consistency | 0.800 | |  | 0.790 | |
| Overall solution coverage | 0.686 | |  | 0.632 | |

Note: ● = the core condition exists, ⊗ = the core condition is absent, ● = the auxiliary condition exists, and ⊗= the auxiliary condition is absent. A blank space indicates that the condition can exist or be absent. RC, Risk communication; DT, degree of trust; RP, risk perception; NE, negative emotions; PCB, protective coping behavior. S1, and S2 represent different configurations.

**Table S5** Robustness Test of ~ECB

| **Conditions** |  | **~ECB** | | | | | |  |
| --- | --- | --- | --- | --- | --- | --- | --- | --- |
|  | **(n=735, cutoff=0.909)** | | | |  | **(n=367, cutoff=0.910)** | | |
|  | **S3** | | **S4a** | **S4b** |  | **S3** | **S4** | **S4b** |
| RC | ⊗ | | ⊗ |  |  | ⊗ | ⊗ | ● |
| DT | ● | |  | ● |  | ● | ⊗ | ● |
| RP |  | | ● | ● |  | ⊗ | ● | ● |
| NE | ⊗ | | ⊗ | ⊗ | ⊗ | ⊗ | ⊗ | ⊗ |
| Consistency | 0.893 | | 0.909 | 0.916 |  | 0.915 | 0.933 | 0.910 |
| Raw coverage | 0.265 | | 0.285 | 0.265 |  | 0.217 | 0.231 | 0.214 |
| Unique coverage | 0.065 | | 0.084 | 0.066 |  | 0.073 | 0.092 | 0.080 |
| Overall solution consistency | 0.869 | | | |  | 0.892 | | |
| Overall solution coverage | 0.415 | | | |  | 0.403 | | |

Note: ● = the core condition exists, ⊗= the core condition is absent, ● = the auxiliary condition exists, and ⊗ = the auxiliary condition is absent. A blank space indicates that the condition can exist or be absent. RC, Risk communication; DT, degree of trust; RP, risk perception; NE, negative emotions; ECB, excessive coping behavior. ~ represent the absence of the condition or outcome. SS3, S4a, and S4b represent different configurations.
